# Impact of Maturity on Volatile Flavor Compounds and Sensory Quality of Distilled Spirit from Hetianhong Table Grapes

**DOI:** 10.3390/foods15173004

**Published:** 2026-08-26

**Authors:** Lei Zhang, Hexiang Bai, Dexin Yang, Mengrong Chen, Xingyuang Yang, Xuewen Li, Qiling Chen

**Affiliations:** College of Food Science and Pharmacy, Xinjiang Agricultural University, Nongda East Road 311, Urumqi 830052, China; eternal-2026@outlook.com (L.Z.); bxh15531946134@outlook.com (H.B.); yangdexin69@outlook.com (D.Y.); acc1408726@163.com (M.C.); 15531946134@163.com (X.Y.);

**Keywords:** Hetianhong table grape, distilled wine, maturity, HS-SPME-GC-MS, volatile flavor compounds

## Abstract

This study examined the effect of grape ripening on the distillate quality of Hetianhong table grapes across three stages of maturity (17, 20, and 24 °Brix). Through the application of HS-SPME-GC-MS, a total of 72 volatile aroma compounds were identified. As the grapes matured from 17 to 24 °Brix, there was an 8.48-fold increase in volatile fatty acids, accompanied by a rise in ester concentrations, while significant reductions were observed in naphthalene derivatives, aldehydes/ketones, and terpenols. Notably, the distillate at 20 °Brix demonstrated the highest concentration of higher alcohols, the richest aroma and superior sensory attributes. The 2-methyl-1-propanol concentration was 15.70 times greater at 20 °Brix than at 24 °Brix, while octanoic acid concentration was 97.93 times higher at 20 °Brix compared to 17 °Brix. Overall, Hetianhong table grapes are ideal for distilled spirits, with optimal maturity modulating the final flavor profile. This research supports value-added processing of these grapes.

## 1. Introduction

Xinjiang is the largest and most productive grape and wine-producing region in China. The Hetianhong grape (*Vitis vinifera* L.), a prominent variety native to southern Xinjiang, is mainly grown in the Hetian region. Its berries are characterized by their thickness and plumpness, abundant juice, with a sugar content exceeding 22 °Brix and moderate acidity, which contributes to a well-balanced sweet-tart taste. Moreover, the relatively large berry size and thick skin enhance their durability for storage and transport [1]. This variety has high tolerance to saline-alkali conditions [2], making it well-suited to the harsh environment of the southern rim of the Tarim Basin. The Hetian Prefecture, where this variety is predominantly cultivated, features a typical inland temperate desert climate, receiving 5800–6200 MJ/m^2^ of annual solar radiation and 2500–3100 h of sunshine per year. The multi-year mean temperature is 12.5 °C, while annual precipitation averages merely 36.4 mm—in stark contrast to an evaporation rate of up to 2618 mm [3]. As the predominant local cultivar, Hetianhong has a planted area exceeding 5600 hectares.

However, the industrial utilization of Hetianhong grapes is predominantly focused on raisin production and the traditional brewing of Musalais [4,5], leaving the scope of application relatively narrow. Musalais is a traditional fermented beverage indigenous to Awat County, Xinjiang. It is often referred to as the “living fossil” of Chinese wine culture, with historical roots tracing back to the Han and Tang dynasties. As the primary raw material for this ancient beverage, Hetianhong grapes share a historical lineage that parallels the antiquity of Musalais itself. The applicability of these grapes in the production of distilled liquor remains largely undetermined, presenting opportunities for diversification into more valuable products.

When the Hetianhong grape berries ripen, their skins show partial and uneven pink discoloration. The maturity of grapes strongly determines the quality of wine and distilled spirits, mainly due to its effect on the chemical composition and sensory characteristics [6]. The volatile aroma compounds that ultimately define wine quality originate from two fundamentally distinct sources: grape-derived (varietal) compounds, which exist in the berry as free volatiles or nonvolatile glycosidic precursors, and fermentation-derived compounds, which are synthesized de novo by yeast during alcoholic fermentation [7,8]. Grape-derived aroma compounds, primarily terpenes, volatile thiols and methoxypyrazines, are directly modulated by grape maturity, vineyard conditions, and postharvest dehydration, all of which influence the biosynthesis, accumulation, and subsequent hydrolytic or enzymatic release of their glycosidically bound precursors [7,9]. In contrast, the quantitatively dominant fermentation-derived volatiles, higher alcohols, acetate and ethyl esters, and medium-chain fatty acids, are products of yeast primary and secondary metabolism. Higher alcohols are synthesized principally via the Ehrlich pathway (catabolism of branched-chain amino acids) and the Harris pathway (de novo synthesis from α-keto acid intermediates of sugar metabolism); acetate esters are formed through alcohol acetyltransferase (AAT)-mediated condensation of acetyl-CoA with higher alcohols; and medium-chain fatty acids are released as intermediates from the fatty acid synthase (FAS) complex under the hypoxic conditions of fermentation [10,11,12]. Critically, the composition and concentration of these fermentation-derived volatiles are not dictated solely by the yeast strain; they are profoundly modulated by the chemical environment of the grape must, including sugar concentration, yeast assimilable nitrogen (YAN) levels and amino acid profiles, lipid content, and pH, all of which undergo dynamic changes during grape ripening [10,13]. Grape maturity therefore exerts a dual influence on wine volatile profiles: it directly determines the pool of grape-derived aroma compounds and their bound precursors, and indirectly shapes the metabolic landscape in which yeast produces fermentation-derived volatiles [14]. Consequently, these interrelated factors further govern the volatile flavor profile of the resulting distilled spirit. Therefore, selecting grapes at the correct stage of maturity are essential for improving the quality of the distilled spirit. Conducting comprehensive studies on these processes can yield novel scientific insights that can improve grape harvesting and promote the production of high-quality alcohol.

In this study, we assessed the applicability of Hetianhong grapes in the production of distilled spirits and determined how grape maturity affects the volatile flavor compounds of the spirits.

## 2. Materials and Methods

### 2.1. Grape Materials

Hetianhong grapes were obtained from Yutian County, Hetian Prefecture, Xinjiang. Approximately 60 vines with uniform growth vigor were selected using a Z-shaped sampling pattern, with border-row vines excluded to minimize edge effect; the selected vines were divided into three groups (~20 vines per group) designated for harvest at target soluble solids contents of 17, 20, and 24 °Brix, respectively. Following the berry enlargement phase, SSC was monitored every 7 days. On each sampling occasion, three replicate samples were collected per group, with each replicate consisting of 100 berries taken from the top, middle, and bottom positions of clusters on both sun-exposed and shaded sides; berries were immediately pressed, and the juice was analyzed for SSC using a handheld digital refractometer (Pocket Refractometer PAL-3, ATAGO Instruments, Tokyo, Japan), as well as total sugar content and total acid content. The monitoring frequency was increased to daily when SSC approached within 1–2 °Brix of the target value. Grapes from each group were hand-harvested upon reaching the respective target SSC (17, 20, and 24 °Brix), immediately transported to the laboratory, and damaged or defective berries were manually removed to ensure sample uniformity.

### 2.2. The Manufacturing Process for Hetianhong Distilled Wine

Hetianhong grapes harvested at three levels of ripeness (17, 20, and 24 °Brix) were destemmed, crushed, and immediately pressed using a small bladder press to obtain clear juice. Next, pectinase (LAFAZYM CL, 60 mg/L) was added, and the must was kept at room temperature for 6–8 h, chilled to 4 °C, and settled for 48 h. To obtain a clear juice, the juice was treated with bentonite for 48 h. After racking into sterilized fermenters, the juice was inoculated with 2 g/L rehydrated Zymaflore^®^ CX9 yeast. Alcoholic fermentation was conducted at 16 °C until the specific gravity dropped to 0.996. The LAFAZYM CL pectinase, bentonite, and Zymaflore^®^ CX9 yeast used in this study were purchased from Laffort (Bordeaux, France).

**Figure 1:**
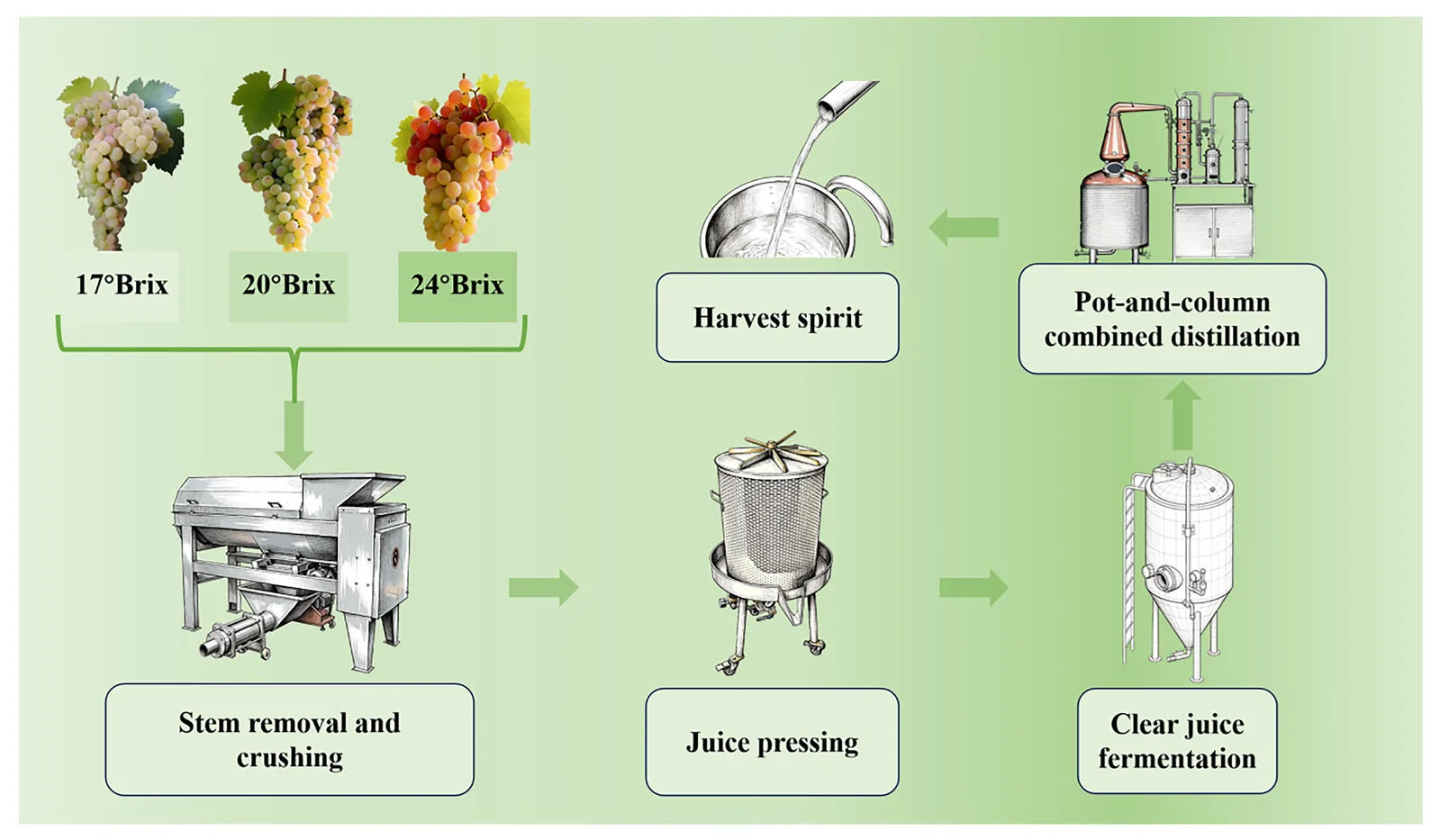
Flowchart of the production process for Hetianhong distilled wine.

The finished wines were distilled using a column still (Zhongniang Intelligent Manufacturing Co., Ltd., Zhengzhou, China). The kettle was heated with steam to 85–90 °C, and the generated vapors ascended the column, where they underwent reflux exchange to concentrate ethanol and aroma compounds. A heads cut of 1‰ (*v*/*v*) was removed to discard methanol and low-boiling compounds. The heart fraction was collected until the alcohol content of the tails dropped to 20% alcohol by volume (ABV), at which point distillation was terminated. The overall workflow is illustrated in Figure 1.

### 2.3. Physicochemical Analysis

The physicochemical indices of the base wine were determined according to the GB/T 15038-2006 standard [15]. The alcoholic strength of the distillates was measured using an alcoholmeter. Reducing sugars, total acidity, and pH were assessed using a Foss WineScan FT120 (Foss, Hillerød, Denmark).

### 2.4. Sensory Evaluation

Quantitative descriptive analysis (QDA) was performed following the protocol described [16], and the detailed procedures were as follows. All sensory analyses were conducted in the sensory laboratory of Xinjiang Agricultural University, equipped with 40 individual evaluation booths, an independent sample preparation room, and controlled lighting and ventilation. Twenty-five senior enology students (aged 19–24 years, senior undergraduate students majoring in enology with 1–3 years of wine sensory evaluation experience) were initially screened for normal olfactory acuity and descriptive ability in accordance with GB/T 16291.1-2012 [17], and subsequently underwent a four-week training program (20–30 h) comprising fundamental knowledge of grape distilled spirits, basic sensory ability training, and identification of sensory attributes with establishment and use of intensity scales. Based on training performance, 18 qualified panelists (8 males, 10 females) were selected as the final descriptive analysis panel. A consensus vocabulary of sensory descriptors was developed by two national Level-1 wine tasters with reference to GB/T 15038-2006 and GB/T 33405-2016 [18], and refined according to the aroma and taste profile of the distillates following four principles: typicality, distinctiveness, standardization, and independence. Samples (20 mL) were presented monadically in ISO-compliant wine-tasting glasses coded with random three-digit numbers at 17 °C. To account for temperature-dependent aroma volatility, two aliquots of each sample were provided per evaluation session. Each session was completed within 1 h, and the entire assessment was repeated three times. Panelists rated the intensity of each descriptor on a 10-point scale (1–3: weak, 4–6: moderate, 7–8: strong, 9–10: very strong). Then, the mean scores for each attribute were calculated for subsequent statistical analyses.

### 2.5. Volatile Compound Profiling of Hetianhong Distilled Wines

Volatile flavor compounds were analyzed via headspace solid-phase microextraction coupled with gas chromatography mass spectrometry (HS-SPME-GC-MS, Agilent 8890–7000e, Agilent Technologies, Inc., Santa Clara, CA, USA) [19]. Wine samples were diluted to 12% (*v*/*v*) ethanol; 5.0 mL of diluted sample, 1.0 g NaCl, and 10.0 µL of 4-methyl-2-pentanol (1.0 g/L, internal standard) were placed in a 20 mL headspace vial, equilibrated at 40 °C for 30 min, and extracted at the same temperature under stirring for 30 min. Desorption was at 250 °C for 8 min. GC separation was carried out using an HP-INNOWAX column (60 m × 250 μm × 0.25 μm) with helium as the carrier gas (1.0 mL/min). The temperature program was as follows: 50 °C (1 min) → 220 °C at 2 °C/min → 280 °C at 5 °C/min, with a post-run at 280 °C for 1 min. MS conditions: EI at 70 eV, m/z 30–350; ion source, transfer line, and quadrupole temperatures were set at 230, 250, and 150 °C, respectively. Compounds were identified by RI and mass spectra (standards or NIST20). Quantification was performed using the external standard calibration method. Calibration curves were constructed in a simulated wine matrix composed of tartaric acid and glucose solution (pH 3.5), with 15 concentration levels prepared for each standard. For compounds lacking authentic reference standards, semi-quantification was performed using structurally analogous compounds. All samples were analyzed in triplicate, and results are reported as mean values. OAV = concentration/threshold.

### 2.6. Data Analysis

The raw data were initially organized using Microsoft Excel and are presented as the mean ± standard deviation (mean ± SD). To visualize the data, we generated radar plots, heatmaps, and principal component analysis (PCA) outputs using Origin 2024. Orthogonal partial least-squares discriminant analysis (OPLS-DA) was conducted using SIMCA 14.1 to calculate variable importance in projection (VIP) values. The data on the volatile compounds were evaluated in SPSS 24.0 by conducting one-way analysis of variance (ANOVA), followed by Duncan’s multiple-range test. Compounds were considered to be differential volatiles when the *p*-value was <0.05 and the VIP value was ≥1.

## 3. Results and Discussion

### 3.1. Comparison Analysis of the Physicochemical Properties of Hetianhong Grapes and Corresponding Distillates Across Various Maturation Stages

During grape ripening, sugars accumulate and organic acids are degraded, while grape-derived volatile aroma compounds are biosynthesized within the berry [20]. Together with must composition parameters, these grape-derived constituents establish the chemical foundation from which the aromatic complexity of distilled spirits subsequently develops through yeast fermentation [21]. Chemical analysis of Hetianhong grapes (Table S1) revealed that as the soluble solids increased from 17 to 24 °Brix, total sugars increased significantly, while total acidity (expressed as sulfuric acid equivalents) decreased. These changes increased the sugar-to-acid ratio from 17.19 to 35.28. Even when the sugar concentration reached 250 g/L, the titratable acidity remained above 7.00 g/L. During the production of distilled spirits, the acidity of grape raw materials plays an important role, as it directly affects the oxidative stability [22], the fermentation process, and the quality of the final product [23]. For Hetianhong grapes, optimal total acidity maintains the flavor balance of the resulting spirits. It not only improves the taste and stability of the product but also inhibits the growth of undesirable microorganisms during fermentation, thereby enhancing product quality and safety [24].

All Hetianhong distillates had an alcohol content ≥ 68%vol at 20 °C (Table S2). Total acidity decreased progressively from 2.45 to 2.13 with the rise in grape ripeness, and volatile acids also declined in content. The residual acidity reduces the pungency of the spirits and promotes the formation of esters. The absorbance at 420 nm ranged steadily from 0.02 to 0.04, indicating that grape maturity had little effect on copper-catalyzed browning and phenolic oxidation. Accordingly, all distillates were clear and colorless, complying with the visual quality standards for high-grade distilled spirits.

### 3.2. Effect of Grape Maturity on the Volatile Compound Profile of Hetianhong Liquor

Grape maturation drives substantial changes in must composition, including sugars, organic acids, amino acids, and yeast assimilable nitrogen (YAN), which collectively modulate yeast metabolic activity and fermentation kinetics, thereby shaping the volatile compound profile of the resulting distilled spirit [25].

**Figure 2:**
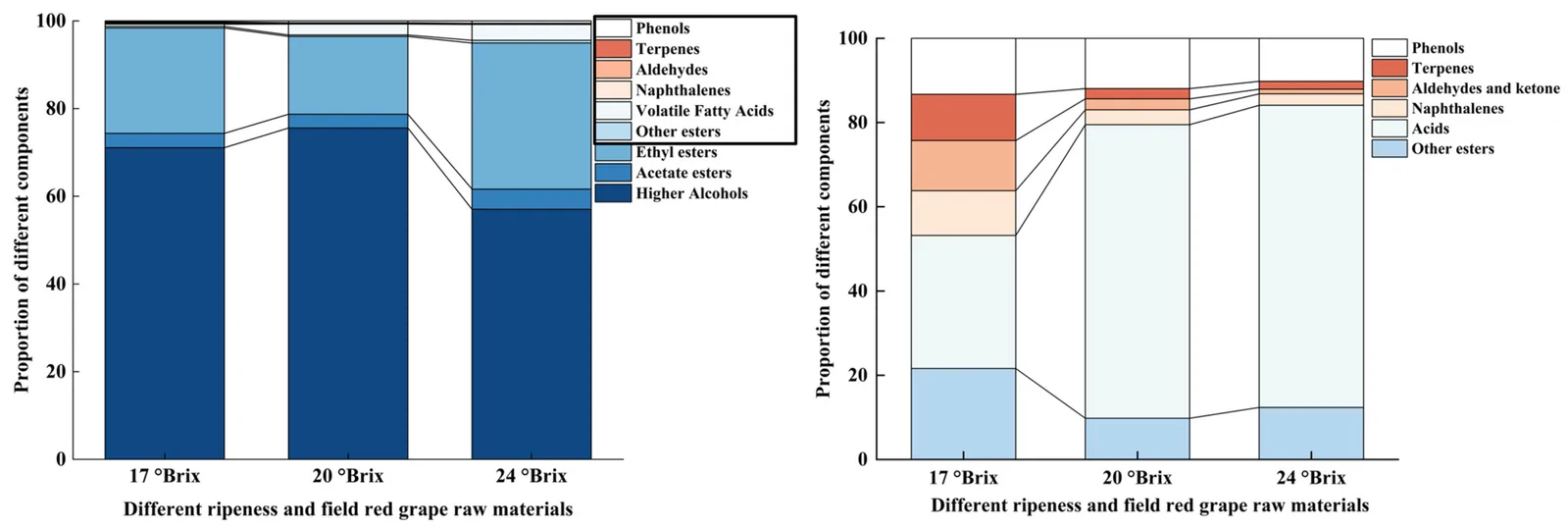
Stacked chart illustrating the total content of different classes of aroma substances in the distilled Hetianhong wines. Each class is distinguished by a unique color.

A total of 72 volatile flavor substances were identified in Hetianhong liquor, comprising 9 higher alcohols, 37 esters (including eight acetic esters, 14 ethyl esters, and 15 other esters), 11 terpenes, 2 aldehydes, 8 naphthalenes, 4 volatile fatty acids, and one phenolic compound (Table 1). With advancing grape maturity, the total concentration of fermentation-derived esters and volatile fatty acids increased significantly (*p* < 0.05), whereas the total concentration of grape-derived naphthalenes, terpenes [26], and aldehyde/ketone compounds exhibited a significant decreasing trend (Table S3). The stacked bar chart (Figure 2) illustrates the variation in the proportions of different classes of volatile flavor compounds within the total flavor content of Hetianhong distilled wines as a function of grape maturity. In the spirit produced from grapes harvested at 20 °Brix, higher alcohols constituted the largest relative proportion of the volatile profile. In addition, in the spirit produced from grapes harvested at 24 °Brix, fermentation-derived esters, particularly ethyl esters, together with volatile fatty acids were more abundant than those in spirits produced from grapes of the other two maturity levels.

**Figure 3:**
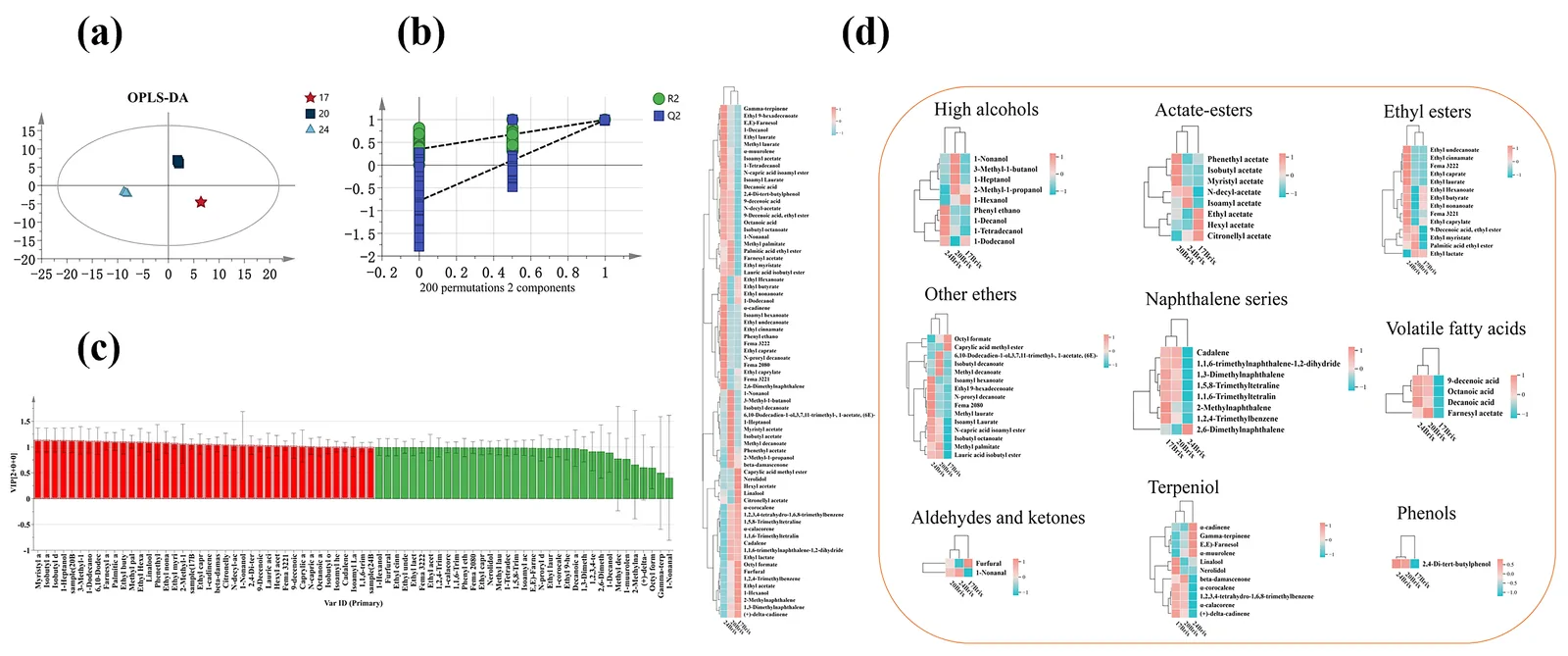
Multivariate statistical analysis of volatile compound profiles in Hetianhong distilled spirits across grape maturity stages. (**a**) OPLS-DA score plot discriminating spirits produced from grapes at three maturity stages (17, 20, and 24 °Brix). (**b**) Permutation test (n = 200) validating model reliability. (**c**) Variable importance in projection (VIP) scores of volatile compounds derived from the OPLS-DA model. (**d**) Hierarchical clustering heatmap of volatile compound profiles.

OPLS-DA effectively discriminated the volatile profiles of Hetianhong distilled spirits across the three maturity stages (Figure 3a), with R^2^X = 0.916, R^2^Y = 0.998, and Q^2^ = 0.994. The 200-permutation test further confirmed model validity, with the Q^2^ regression line intersecting below zero (Figure 3b). Among the 72 volatile compounds subjected to modeling, 37 with VIP > 1 were designated as key differential markers (Figure 3c). Hierarchical cluster analysis of these 72 compounds (Figure 3d) revealed category-dependent clustering patterns: higher alcohols and ethyl esters from the 17 and 20 °Brix groups clustered together, separating from the 24 °Brix group, whereas the remaining compounds grouped the 20 and 24 °Brix samples together, distinct from the 17 °Brix group.

#### 3.2.1. Higher Alcoholic Compounds of Hetianhong Liquor at Different Stages of Maturation

Higher alcohols, like aliphatic and aromatic alcohols, are major by-products of yeast metabolism, particularly produced during fermentation processes [27]. These compounds, often referred to as fusel alcohols, are produced through the catabolism of amino acids via the Ehrlich pathway. In this process, amino acids are first transaminated to α-keto acids, which are then decarboxylated and reduced to form higher alcohols [28]. Various factors, including the yeast strain used [29], the fermentation conditions, and the availability of precursors such as amino acids [30], influence the production of these alcohols.

In this study, higher alcoholic compounds were the most abundant volatile compounds in all Hetianhong liquors (Figure 2 and Table S3). The total higher alcohol content in the Hetianhong liquor treated at 20 °Brix was approximately 1.5 times greater than that observed in the Hetianhong liquor treated at 17 and 24 °Brix (*p* < 0.05).

Among the nine higher alcohols identified (Table S4), 3-methyl-1-butanol had the highest concentration, followed by 2-methyl-1-propanol, phenylethanol, and 1-Hexanol. 3-methyl-1-butanol, known for its banana and fruity-floral aromas [31,32], was most concentrated in the 20 °Brix group. The concentration of 2-methyl-1-propanol, whose formation is tightly coupled to valine availability and yeast metabolic status [33,34], was also the highest in the 20 °Brix group, being 15.70 times higher than in the 24 °Brix group. The concentration of phenylethanol, a rose- and honey-like aromatic higher alcohol derived from phenylalanine catabolism [35,36], was the highest in the 24 °Brix group. Moreover, the concentration of 1-Hexanol was considerably lower in spirits produced from more mature grapes.

#### 3.2.2. Ester Compounds of Hetianhong Liquor at Different Maturation Stages

Ester compounds constituted the second most abundant volatile group in Hetianhong distillates, following higher alcohols, with total concentrations increasing markedly at advanced grape maturity stages (Figure 2 and Table S3).

Among the eight identified acetate esters, the concentration of isoamyl acetate was the highest, followed by the concentration of phenylethyl acetate, decyl acetate, and hexyl acetate (Table S4). The concentrations of both isoamyl acetate and decyl acetate increased significantly as the grapes matured. The concentration of isoamyl acetate in the 24 °Brix group was approximately double that observed in the 17 °Brix group. The concentrations of phenylethyl acetate, isobutyl acetate, and tetradecanoyl acetate were significantly higher in the 20 °Brix group than in the 17 and 24 °Brix groups. In contrast, the concentration of ethyl acetate decreased as the grapes ripened.

Among the 14 ethyl esters identified, the predominant compounds were ethyl decanoate, ethyl octanoate, ethyl laurate, ethyl 9-decenoate, ethyl hexanoate, ethyl butyrate, and ethyl lactate. The concentration of ethyl 9-decenoate increased continuously and significantly as ripeness increased, while the concentrations of ethyl decanoate, ethyl octanoate, ethyl hexanoate, and ethyl butyrate first decreased and then increased throughout the ripening process.

Decanoic-3-methyl-butyl ester and octanoic-3-methyl-butyl ester were the predominant non-acetate/ethyl esters, and their concentrations increased significantly as the Hetianhong grapes ripened.

Esters are key contributors to the fruity and floral aroma signatures of distilled spirits [37]. As fermentation-derived compounds, their biosynthesis is intimately linked to yeast metabolism, specifically, the esterification of ethanol with acyl-CoA intermediates generated from sugar and lipid catabolism [38,39], as well as chemical esterification during aging [40]. The observed ester profiles across maturity stages therefore reflect the indirect influence of grape maturity on fermentation substrate composition, which in turn modulates yeast ester-synthesizing activity.

#### 3.2.3. Volatile Fatty Acids in Hetianhong Distilled Wines at Different Maturation Stages

Volatile fatty acids (VFAs) play an important role in the production and sensory characteristics of distilled wine, contributing to the aroma and flavor profiles [41]. In this study, the total content of VFAs increased significantly as the grapes became more mature (Table S3). The total content of VFAs was 8.48 times greater in the 24 °Brix group than in the 17 °Brix group. The concentration of octanoic acid was 97.93 times higher in the 20 °Brix group than in the 17 °Brix group. Moreover, it was 115.98 times higher in the 24 °Brix group than in the 17 °Brix group. The concentration of decanoic acid in the 20 °Brix treatment group was 3.63 times higher than that in the 17 °Brix group, whereas the 24 °Brix group contained 5.33 times the concentration of decanoic acid found in the 17 °Brix group (Table S4).

Medium-chain fatty acids (MCFAs) such as octanoic acid (C8) and decanoic acid (C10) are produced by *Saccharomyces cerevisiae* as side-products of the fatty acid synthase (FAS) complex during alcoholic fermentation [42,43]. The yeast FAS complex normally elongates the acyl chain to C16 (palmitic acid) or C18 (stearic acid) before the thioesterase domain catalyzes product release; however, under fermentation stress conditions, including elevated osmotic pressure, nitrogen limitation, and altered carbon-to-nitrogen ratios, the processivity of the FAS complex can be compromised, leading to premature release of intermediate-chain-length acyl groups as free MCFAs [44]. The preferential accumulation of octanoic acid (C8) over decanoic acid (C10) at higher grape maturity levels suggests that the compositional changes in the fermentation substrate associated with ripening (e.g., increased sugar concentration, altered amino acid and lipid profiles) may differentially affect the efficiency of fatty acid chain elongation and the balance between de novo FAS activity and exogenous lipid utilization by yeast [45]. Specifically, the marked increase in octanoic acid may reflect a greater sensitivity of the C8 → C10 elongation step to fermentation conditions, rather than a shift in the intrinsic product specificity of the FAS thioesterase domain.

Research on VFAs in distilled wines is less extensive than that in regular wines. VFAs significantly influence the taste and aroma of wines by interacting with other components [46]. High lipid levels affect the formation of higher alcohols and esters during the fermentation of white wines, highlighting the complex relationship between VFAs and other volatile compounds [47]. Adjusting unsaturated VFAs during fermentation alters the production of acetate and ethyl ester [48]. Collectively, the present findings demonstrate that grape maturity indirectly shapes the VFA profile of Hetianhong distillates, principally by altering the lipid and nitrogenous composition of the fermentation must, which in turn governs yeast fatty acid metabolism during fermentation.

#### 3.2.4. Terpenoid Compounds in Hetianhong Liquor at Different Stages of Maturation

Terpenoids constitute a diverse class of naturally occurring organic chemicals derived from terpenes. They play a significant role in the aroma and flavor profiles of distilled wines and spirits. Terpenes dominate during early grape development, whereas benzene derivatives such as phenylethanol and 2-phenylacetaldehyde become more prominent in later phases of maturation [49]. Besides imparting the signature aromas of spirits, they significantly influence the sensory profile of these spirits [30].

The overall concentration of terpenoid compounds in distilled wine decreased significantly as the grape raw material matured. The concentrations of nerol and α-dehydro-*β*-ionone decreased substantially as the grapes matured (Table S4). In contrast, the concentration of damascenone was significantly higher in the 20 °Brix group than in the other groups. Linalool demonstrated a distinct pattern, where its concentration first decreased significantly and then increased significantly.

The sensory impact of terpenoids in alcoholic beverages is governed not only by their absolute concentrations but also by matrix effects that modulate their volatility and perception. Non-volatile matrix constituents can substantially enhance terpenoid headspace volatility, thereby amplifying their aromatic expression beyond what concentration data alone would predict [50]. Furthermore, both the choice of distillation technique [51] and the fermentation strain employed [52] critically influence the extent to which glycosidically bound terpenoid precursors are hydrolyzed and released into the final spirit. Collectively, the present findings indicate that grape maturity directly governs the terpenoid precursor pool available in the must, while downstream processing factors, including fermentation and distillation, determine the ultimate terpenoid profile perceived in the finished distillate.

#### 3.2.5. Naphthalene Compounds and Their Derivatives in Hetianhong Liquor at Different Stages of Maturation

The overall concentration of naphthalene derivatives in Hetainhong distilled wine decreased significantly as the grapes became more mature (Table S3). Among the eight naphthalene compounds identified, the concentration of 1,3-dimethylnaphthalene was the highest, followed by the concentrations of 2,6-dimethylnaphthalene and juniper naphthalene (Table S4). Moreover, the concentrations of most naphthalene compounds decreased considerably as the Hetianhong grapes became more mature.

The Hetianhong distilled wine had no detectable levels of phthalate esters (PEAs), a class of compounds that are stringently regulated as they pose health risks [53].

#### 3.2.6. Aldehydes, Ketones, and Their Derivatives in Hetianhong Liquor at Different Stages of Maturation

Aldehydes and ketones are important compounds in the flavor profile of distilled wines and contribute significantly to the aromatic complexity and sensory characteristics of wines. In Baijiu, a traditional Chinese liquor, aldehydes are among the key volatile compounds that contribute to its characteristic pungency [54].

These compounds are formed during fermentation and aging and are influenced by various factors, including the type of raw materials used, fermentation conditions, and aging techniques [55,56,57]. The presence and concentration of aldehydes and ketones can strongly affect the overall quality and consumer acceptance of distilled wines.

The overall concentration of aldehydes and ketones in Hetianhong distilled wines decreased significantly as the grape raw material became more mature (Table S3). The primary aldehydes and ketones identified were furfural and nonanal (Table S4). The concentration of furfural, which predominantly imparts a caramel flavor, decreased with maturity. Its concentration at 17 °Brix was 2.95 times higher than that observed at 24 °Brix.

**Table 1:** Analysis of volatile flavor compounds (all tabulated data with OAV > 1) in grape distilled spirits derived from Hetianhong red grapes at different levels of maturity.

CAS	Name	RI	Threshold (μg/L)	OAV			*p*-Value
				17 °Brix	20 °Brix	24 °Brix	
78-83-1	2-Methyl-1-propanol	1480.17	1300 ^A^	156.40	270.00	9.96	0.00
123-51-3	3-Methyl-1-butanol	2063.55	6500 ^A^	88.10	119.56	92.76	0.00
111-70-6	1-Heptanol	2157.85	2450 ^B^	8.20	52.87	8.79	0.00
123-92-2	Isoamyl acetate	1576.59	200 ^A^	125.52	169.06	251.01	0.00
142-92-7	Hexyl acetate	2080.45	670 ^A^	3.86	2.63	2.45	0.00
112-17-4	*N*-decyl-acetate	1566.79	225 ^B^	8.46	14.29	15.00	0.00
103-45-7	Phenethyl acetate	2250.29	650 ^A^	21.71	30.10	18.61	0.00
105-54-4	Ethyl butyrate	1032.04	150 ^A^	2831.31	1919.28	3187.88	0.00
123-66-0	Ethyl Hexanoate	2070.88	76 ^A^	3091.68	1785.60	3589.74	0.00
106-32-1	Ethyl caprylate	916.77	240 ^A^	3599.80	2788.90	4561.68	0.00
110-38-3	Ethyl caprate	2236.21	1100 ^A^	138.46	145.57	237.86	0.17
627-90-7	Ethyl undecanoate	1368.76	0.3 ^D^	389.60	389.77	439.23	0.95
106-33-2	Ethyl laurate	2273.09	640 ^A^	26.61	34.16	73.63	0.04
103-36-6	Ethyl cinnamate	1418.03	48 ^B^	0.21	0.10	9.59	0.00
110-42-9	Methyl decanoate	1353.06	4.3 ^B^	30.56	32.79	30.78	0.20
2035-99-6	3-methylbutyl ester	1364.65	600 ^A^	1.49	1.98	6.10	0.05
111-82-0	Methyl laurate	1663.67	1.5 ^D^	5.73	5.97	7.12	0.00
475-03-6	1,1,6-Trimethyltetralin	1851.74	20 ^B^	1.63	1.48	0.41	0.00
30364-38-6	1,1,6-trimethylnaphthalene-1,2-dihydride	1867.54	2.5 ^B^	18.52	19.20	7.46	0.17
581-42-0	2,6-Dimethylnaphthalene	1724.06	10 ^B^	79.17	79.15	79.20	0.32
575-41-7	1,3-Dimethylnaphthalene	2046.39	42 ^B^	19.01	18.98	18.92	0.06
91-57-6	2-Methylnaphthalene	1687.49	3 ^B^	7.89	7.53	7.37	0.30
124-19-6	1-Nonanal	1111.01	5.8 ^E^	3.38	4.09	4.33	0.58
78-70-6	Linalool	2226.96	6 ^C^	10.13	7.90	8.62	0.00
23726-93-4	beta-damascenone	1680.05	0.002 ^F^	10,780.00	12,820.00	7875.00	0.00
124-07-2	Octanoic acid	2312.48	15,000 ^A^	0.01	1.41	1.67	0.00
334-48-5	Decanoic acid	2348.6	8000 ^A^	0.49	1.79	2.63	0.00

Note: Values within the same column followed by different superscript letters (A–F) indicate significant differences (*p* < 0.05). The threshold represents the threshold acquired in the references [58].

### 3.3. Effect of the Level of Maturity on the Sensory Profile of Hetianhong Distilled Spirit

**Figure 4:**
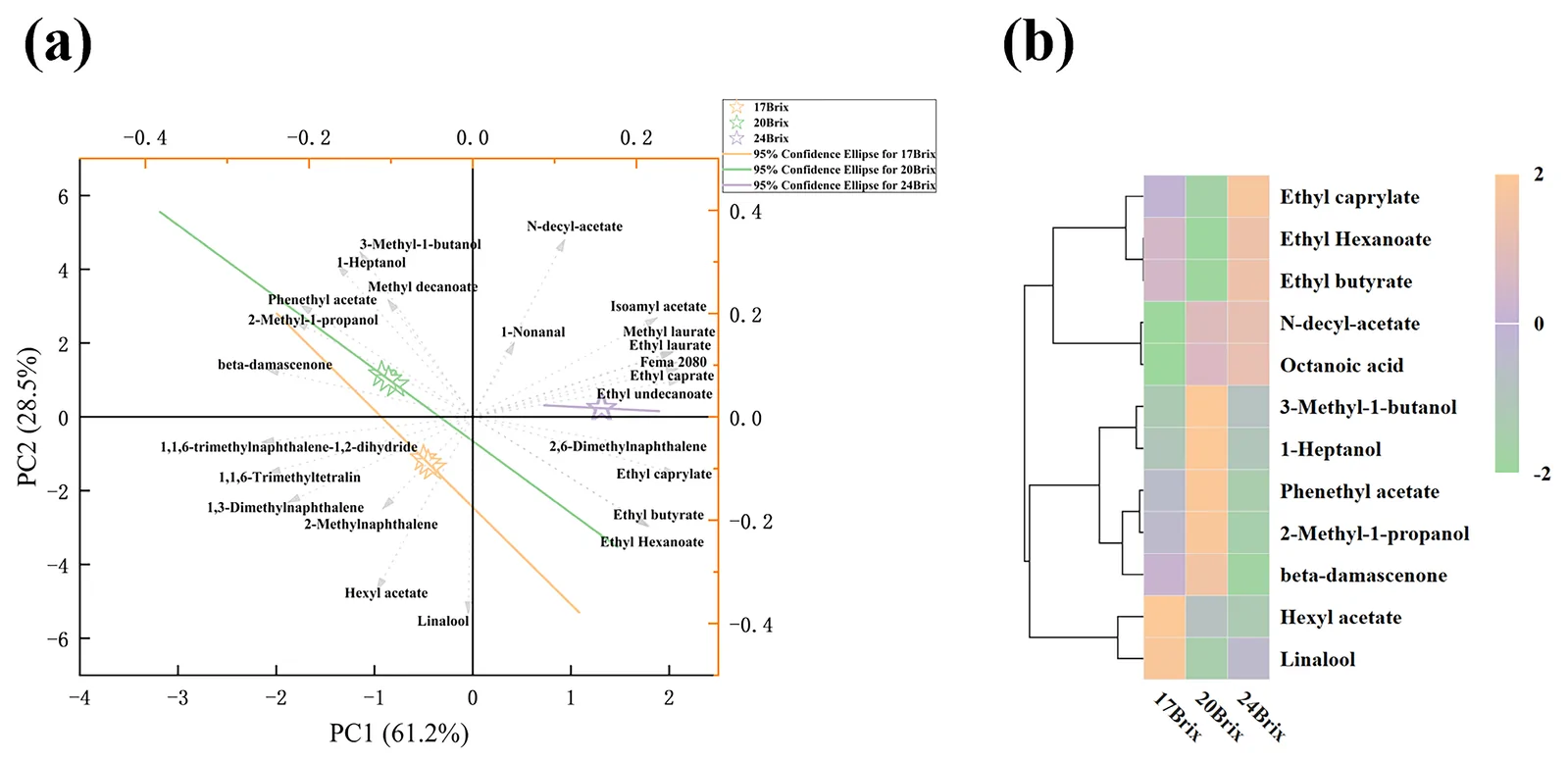
Discrimination and visualization of key aroma-active volatile compounds in Hetianhong distilled spirits across grape maturity stages. (**a**) PCA score plot based on volatile compounds with OAV > 1 in spirits produced from grapes at three maturity stages (17, 20, and 24 °Brix). (**b**) Hierarchical clustering heatmap of key differential volatile compounds screened by OAV > 1, VIP > 1, and *p* < 0.05.

Twenty-seven volatile flavor compounds with odor activity values (OAVs) exceeding 1 were identified and subsequently subjected to principal component analysis (PCA) to delineate their discriminatory capacity across grape maturity stages (Table 1). The PCA model (Figure 4a) captured 89.7% of the total variance, effectively resolving the flavor profile differentiation among samples produced from grapes harvested at 17, 20, and 24 °Brix. Isoamyl acetate and ethyl laurate, both fermentation-derived esters, exhibited a strong positive correlation with the samples in the 20 °Brix group, contributing to the fresh floral and fruity aroma of these samples. 3-methyl-1-butanol, a fermentation-derived higher alcohol, and damascenone, an important norisoprenoid constituent in grape berries and wines [59], were positively associated with the 20 °Brix group, contributing alcoholic and elegant floral notes. Aromatic compounds resembling naphthalene, which impart woody and delicate floral characteristics, were unique to the low-sugar (17 °Brix) sample, suggesting their preferential accumulation at early maturity. Ethyl hexanoate and ethyl octanoate, both fermentation-derived ethyl esters associated with rich fermented fruity aroma, demonstrated a strong positive correlation with the high-sugar (24 °Brix) sample.

To further refine the identification of aroma-active differential markers, hierarchical cluster analysis (HCA; Figure 4b) was performed on the subset of compounds concurrently satisfying VIP > 1, OAV > 1, and *p* < 0.05 (Table S5), representing volatile compounds that are both statistically discriminative and sensorially significant. The 24 °Brix group was distinguished by maximum accumulation of fermentation-derived ethyl esters, particularly ethyl caprylate and ethyl hexanoate. The 20 °Brix group exhibited peak levels of damascenone, phenylethyl acetate, 3-methyl-1-butanol, 1-heptanol, and 2-methyl-1-propanol. The 17 °Brix group, by contrast, was characterized by elevated levels of hexyl acetate and linalool.

**Figure 5:**
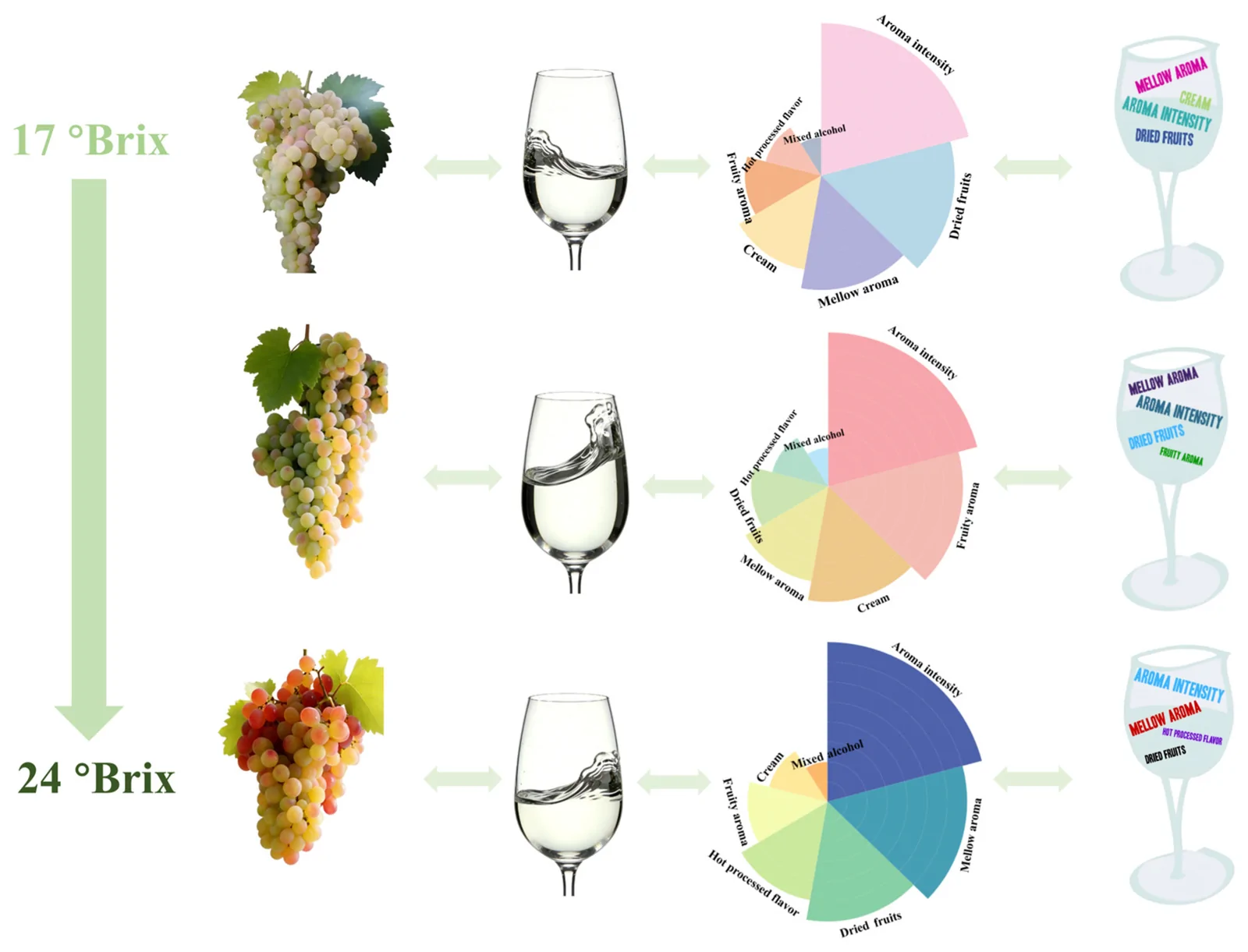
A comprehensive visualization encompassing the three stages of Hetianhong grape maturity, the principal aroma descriptors of the distilled wine, an aroma rose diagram, and the color profile of the spirit. In Column 1, grapes are illustrated at three discrete stages of maturity, characterized by a progressively increasing distribution of pink coloration on the skin as the grapes mature. Column 2 displays the visual color appearance of the distilled spirit. Column 3 presents an aroma rose diagram that illustrates the intensity distribution of each descriptor. Finally, Column 4 highlights the key aroma descriptors identified in the distilled spirit.

The sensory evaluation (Figure 5) was conducted to corroborate the chemical differentiation observed above. In line with the volatile profiles, the 20 °Brix distillate exhibited the most balanced and harmonious aroma, characterized by pronounced floral and fruity notes, primarily attributable to damascenone and phenylethyl acetate, complemented by creamy and mellow undertones derived from 3-methyl-1-butanol and certain esters, alongside well-integrated dried fruit nuances. In contrast, the distillate at 17 °Brix, which was enriched in linalool and hexyl acetate, presented a fresh, green, and citrus character but lacked the intensity of fruity and creamy notes, resulting in a thinner aromatic layer and insufficient richness. The distillate at 24 °Brix, characterized by elevated levels of ethyl esters and octanoic acid, displayed a baked character with increased fusel and solvent off-notes, diminished dried fruit and creamy notes, and a complete loss of freshness. In summary, within the tested maturity range (17–24 °Brix), the 20 °Brix treatment showed the best performance in enhancing desirable aromas, balancing dried fruit and mellowness, and minimizing off-flavors, thus achieving superior quality relative to the other two maturity levels.

## 4. Conclusions

This study demonstrates that Hetianhong table grapes, indigenous to Xinjiang and distinguished by their high acidity, present promising potential as a raw material for spirit production. Grape maturity, through its modulation of must composition (sugars, acidity, amino acid profiles, and yeast assimilable nitrogen, among others), serves as a critical factor that shapes yeast metabolism during fermentation and consequently determines the volatile compound profile and sensory attributes of the resulting distillate. By profiling 72 volatile compounds and applying OPLS-DA multivariate analysis, we successfully discriminated Hetianhong distilled spirit samples produced from grapes at different maturity stages.

As grape maturity advanced, the total concentration of fermentation-derived volatile fatty acids increased 8.48-fold from 17 °Brix to 24 °Brix, with a concurrent rise in ester concentrations. Conversely, the total concentrations of grape-derived naphthalene derivatives, aldehydes/ketones, and terpenols significantly decreased. Notably, the spirit produced from grapes harvested at 20 °Brix showed the highest concentrations of higher alcohols, the greatest volatile aroma content, and the most favorable sensory profile.

Among individual compounds, the concentration of 2-methyl-1-propanol was 15.70 times greater at 20 °Brix than at 24 °Brix. Furthermore, the concentrations of phenylethyl acetate, isobutyl acetate, and tetradecanoyl acetate were significantly elevated in the 20 °Brix group compared to the 17 and 24 °Brix groups. Further, the concentration of damascenone was also significantly higher in the 20 °Brix group relative to the other groups. The content of octanoic acid was 97.93 times higher at 20 °Brix and 115.98 times higher at 24 °Brix compared to its concentration at 17 °Brix. Moreover, sensory evaluation indicated that the distillate produced at 20 °Brix exhibited the best performance in enhancing desirable aromas (creamy and fruity notes), balancing dried fruit and mellowness.

Future targeted analyses of must nitrogen and lipid substrate dynamics across maturity stages are needed to elucidate the mechanistic basis by which grape maturity governs fermentation-derived volatile formation. Collectively, these findings provide theoretical insights for optimizing table grape utilization in spirit production and establish a scientific foundation for the standardization of production processes and quality control in grape distilled spirits.

## Data Availability

The original contributions presented in the study are included in the article/Supplementary Material, further inquiries can be directed to the corresponding author.

## Supplementary Materials

The following supporting information can be downloaded at: https://www.mdpi.com/article/10.3390/foods15173004/s1, Table S1: The physicochemical properties of Hetianhong grapes at three levels of maturity are presented; Table S2: Physicochemical properties of distilled wines made from Hetianhong grapes at three levels of maturity are presented; Table S3: The total content of volatile flavor compound classes in Hetianhong distilled wines from raw materials at different levels of maturity is presented; Table S4: Analysis of volatile flavor compounds in grape distilled spirits derived from Hetianhong red grapes at different levels of maturity; Table S5: Key differential volatile aroma compounds screened by OAV > 1, VIP > 1, and *p* < 0.05.
