# Supplementary material for: Impact of Maturity on Volatile Flavor Compounds and Sensory Quality of Distilled Spirit from Hetianhong Table Grapes
Source: Foods. 2026 Aug 26;15(17):3004. doi: 10.3390/foods15173004 (PMC13564833; doi:10.3390/foods15173004)
Supplement: Supplementary file 1 [file foods-15-03004-s001.zip › foods-4420003-supplementary.pdf]

Table S1. The physicochemical properties of Hetianhong grapes at three levels of maturity are presented.

Soluble solid content	Total Acid (g/L)	Reducing sugar (g/L)	Sugar/Acid ratio	pH
17 °Brix	10.77±0.00[a]	185.17±0.25[c]	17.19	3.24±0.01[c]
20 °Brix	8.46±0.00[b]	216.60±0.10[b]	25.60	3.58±0.02[b]
24 °Brix	7.15±0.06[c]	252.27±5.70[a]	35.28	3.82±0.01[a]

Note: Values within the same column followed by different superscript letters (a, b, and c) indicate significant differences ($p < 0.05$).

Table S2. Physicochemical properties of distilled wines made from Hetianhong grapes at three levels of maturity are presented.

Raw material maturity	Alcohol content (% vol)	Total acidity (g/L)	Volatile acid (g/L)	Total sugar (g/L)	Absorbance (420 nm)
17 °Brix	70±0.15	2.45±0.05	0.26±0.01	1.33±0.06	0.02±0.00
20 °Brix	70±0.01	2.27±0.06	0.24±0.01	1.82±0.08	0.04±0.00
24 °Brix	68±0.10	2.13±0.06	0.24±0.01	1.73±0.06	0.02±0.00

Table S3. The total content of volatile flavor compound classes in Hetianhong distilled wines from raw materials at different levels of maturity is presented.

Raw material maturity	High alcohols (μg/L)	Esters			Volatile fatty acids (μg/L)	Naphthalene series (μg/L)	Aldehydes and ketones (μg/L)	Terpeniol (μg/L)	Phenols (μg/L)
		Actate-esters (μg/L)	Ethyl esters (μg/L)	Other ethers (μg/L)					
17 °Brix	791,002.66±8166.09[b]	36,031.91±110.47[c]	267,481.16±7345.29[b]	3,869.28±88.07[c]	5,653.77±917.38[c]	1,903.03±0.96[a]	2,129.94±51.88[a]	1,965.59±1.25[a]	2,376.53±47.53[b]
20 °Brix	1,142,224.93±12951.79[a]	47,482.22±1351.29[b]	268,256.72±10941.20[b]	5,240.50±231.02[b]	37,281.22±5572.58[b]	1,887.11±0.09[b]	1,391.70±61.07[b]	1,304.32±0.90[b]	6,384.31±75.05[a]
24 °Brix	754,286.56±5442.92[c]	60,755.19±181.64[a]	440,415.85±7569.59[a]	8,262.48±65.34[a]	47,944.17±975.07[a]	1,797.80±0.95[c]	740.74±20.09[c]	1,251.30±0.07[c]	6,821.96±685.58[a]

Note: The data are presented as the mean ± SD (n = 3). Values within the same column followed by different superscript letters (a, b, and c) indicate significant differences ($p < 0.05$).

Table S4. Analysis of volatile flavor compounds in grape distilled spirits derived from Hetianhong red grapes at different levels of maturity.

CAS	Name	Category	RI	17°Brix (μg/L)	20°Brix (μg/L)	24°Brix (μg/L)	Threshold (μg/L)	OAV			*p*-value
								17°Brix	20°Brix	24°Brix	
78-83-1	2-Methyl-1-propanol	A1	1480.17	203300.37±261.93^{b}	351003.71±35793.20^{a}	12948.07±22338.11^{c}	1300	156.40	270.00	9.96	0.00
123-51-3	3-Methyl-1-butanol	A2	2063.55	572649.35±8084.63^{b}	777111.00±22193.26^{a}	602942.85±18077.46^{b}	6500	88.10	119.56	92.76	0.00
111-70-6	1-Heptanol	A3	2157.85	44.26±0.87^{b}	285.50±12.62^{a}	47.48±2.11^{b}	2450	8.20	52.87	8.79	0.00
143-08-8	1-Nonanol	A4	1557.53	44.26±0.12^{b}	45.86±0.72^{a}	44.54±0.21^{b}	310	0.97	1.01	0.98	0.02
112-30-1	1-Decanol	A5	2240.46	33.16±4.97^{b}	35.66±0.68^{b}	45.68±1.86^{a}	210	0.16	0.17	0.22	0.44
60-12-8	Phenyl ethano	A6	2292.79	10155.85±135.93^{b}	10342.20±612.66^{b}	19376.65±1194.63^{a}	—	—	—	—	0.63
112-72-1	1-Tetradecanol	A7	1779.87	146.33±0.19^{c}	153.24±0.49^{b}	164.92±0.85^{a}	500	0.29	0.31	0.33	0.00
112-53-8	1-Dodecanol	A8	1716.5	822.94±1.31^{b}	800.43±0.02^{c}	827.16±0.28^{a}	1000	0.82	0.80	0.83	0.00
111-27-3	1-Hexanol	A9	2139.77	3806.14±41.69^{a}	2447.34±20.96^{b}	1355.19±9.17c	5200	0.73	0.47	0.26	0.00
141-78-6	Ethyl acetate	B1	1056.12	466.17±15.82^{a}	376.16±4.83^{b}	316.57±0.96^{c}	17000	0.03	0.02	0.02	0.00
110-19-0	Isobutyl acetate	B2	1291.59	255.84±9.69^{c}	479.44±4.12^{a}	276.78±2.16^{b}	3400	0.08	0.14	0.08	0.00
123-92-2	Isoamyl acetate	B3	1576.59	25103.09±196.32^{c}	33812.85±730.39^{b}	50202.35±133.24^{a}	200	125.52	169.06	251.01	0.00
142-92-7	Hexyl acetate	B4	2080.45	2585.86±44.11^{a}	1761.65±99.21^{b}	1639.43±54.47^{b}	670	3.86	2.63	2.45	0.00
150-84-5	Citronellyl acetate	B5	1857.52	152.25±0.21^{a}	150.44±0.00^{b}	151.31±0.61^{b}	1000	0.15	0.15	0.15	0.00
112-17-4	N-decyl-acetate	B6	1566.79	1903.35±34.64^{c}	3215.63±1.74^{b}	3375.51±67.51^{a}	225	8.46	14.29	15.00	0.00
103-45-7	Phenethyl acetate	B7	2250.29	5418.27±69.80	7513.72±510.45	4644.13±172.59	650	21.71	30.10	18.61	0.00
638-59-5	Myristyl acetate	B8	1999.61	147.08±0.20^{c}	172.34±0.55^{a}	149.09±0.05^{b}	—	—	—	—	0.00
105-54-4	Ethyl butyrate	C1	1032.04	2548.18±13.21^{b}	1727.35±41.94^{c}	2869.09±10.45^{a}	150	2831.31	1919.28	3187.88	0.00
123-66-0	Ethyl Hexanoate	C2	2070.88	15458.41±87.40^{b}	8927.99±571.90^{c}	17948.68±42.80^{a}	76	3091.68	1785.60	3589.74	0.00
97-64-3	Ethyl lactate	C3	2092.48	1849.39±235.80^{a}	2010.86±95.74^{a}	567.60±0.31^{b}	14000	0.04	0.04	0.01	0.33
106-32-1	Ethyl caprylate	C4	916.77	69476.15±199.57^{b}	53825.81±1797.31^{c}	88040.39±707.40^{a}	240	3599.80	2788.90	4561.68	0.00
123-29-5	Ethyl nonanoate	C5	2219.4	133.29±0.19^{b}	86.73±0.37^{c}	162.64±2.29^{a}	850	0.35	0.23	0.43	0.00
110-38-3	Ethyl caprate	C6	2236.21	152308.16±4503.08^{b}	160123.91±6860.84^{b}	261646.40±4471.44^{a}	1100	138.46	145.57	237.86	0.17
627-90-7	Ethyl undecanoate	C7	1368.76	116.88±0.40^{b}	116.93±1.40^{b}	131.77±0.35^{a}	0.3	389.60	389.77	439.23	0.95
106-33-2	Ethyl laurate	C8	2273.09	17030.70±2529.85^{b}	21864.86±940.61^{b}	47120.83±2320.20^{a}	640	26.61	34.16	73.63	0.04
124-06-1	Ethyl myristate	C9	1410.9	153.32±0.02^{b}	505.68±18.64^{a}	451.72±44.90^{a}	5700	0.03	0.09	0.08	0.00
103-36-6	Ethyl cinnamate	C10	1418.03	3.62±0.39^{b}	1.76±0.25^{b}	162.95±22.09^{a}	48	0.21	0.10	9.59	0.00
5457-70-5	Fema 3222	C11	1896.6	156.64±1.49^{b}	161.28±0.23^{b}	231.78±5.16^{a}	10000	0.02	0.02	0.02	0.01
6290-37-5	Fema 3221	C12	1783.92	162.66±1.20^{b}	153.40±0.11^{c}	190.83±0.54^{a}	—	—	—	—	0.00
628-97-7	Palmitic acid ethyl ester	C13	1456.49	261.41±13.15^{c}	520.406±6.87^{a}	405.48±23.17^{b}	14000	0.02	0.04	0.03	0.00
67233-91-4	9-Decenoic acid, ethyl ester	C14	1366.86	7822.36±362.33^{c}	18229.74±810.90^{b}	20485.70±171.07^{a}	—	—	—	—	0.00
54546-22-4	Ethyl 9-hexadecenoate	D1	1472.69	146.18±3.54^{c}	180.25±6.09^{b}	281.10±17.19^{a}	—	—	—	—	0.00

111-11-5	Caprylic acid methyl ester	D2	2155.23	55.55±1.15^{a}	46.36±2.09^{b}	46.65±0.30^{b}	200	0.28	0.23	0.23	0.00
2198-61-0	Isoamyl hexanoate	D3	2168.87	62.42±3.14^{b}	56.91±1.92^{c}	125.78±0.44^{a}	1400	0.04	0.04	0.09	0.06
1300784	Isobutyl octanoate	D4	1251.93	94.19±3.68^{b}	138.30±5.42^{a}	149.80±8.56^{a}	—	—	—	—	0.00
112-32-3	Octyl formate	D5	1510.49	18.88±0.68	17.52±3.12	16.27±0.09	—	—	—	—	0.50
110-42-9	Methyl decanoate	D6	1353.06	131.39±5.83	140.98±9.22	132.35±0.28	4.3	30.56	32.79	30.78	0.20
2035-99-6	Fema 2080	D7	1364.65	893.79±82.57^{c}	1189.17±160.20^{b}	3657.39±0.96^{a}	600	1.49	1.98	6.10	0.05
30673-60-0	N-proryl decanoate	D8	1573.49	12.78±0.03^{b}	13.32±0.18^{b}	17.83±0.62^{a}	—	—	—	—	0.01
30673-38-2	Isobutyl decanoate	D9	1589.32	183.78±0.07^{c}	233.80±2.98^{a}	194.17±3.78^{b}	—	—	—	—	0.00
111-82-0	Methyl laurate	D10	1663.67	8.60±0.02^{c}	8.96±0.04^{b}	10.68±0.05^{a}	1.5	5.73	5.97	7.12	0.00
2306-91-4	N-capric acid isoamyl ester	D11	1682.95	1646.36±196^{c}	2516.21±57.67^{b}	2936.31±52.74^{a}	5000	0.33	0.50	0.59	0.00
37811-72-6	Lauric acid isobutyl ester	D12	1974.59	146.48±0.23^{b}	151.54±0.18^{a}	151.14±1.55^{a}	—	—	—	—	0.00
6309-51-9	Isoamyl Laurate	D13	1765.45	165.17±2.47^{c}	196.51±0.34^{b}	214.55±4.46^{a}	100000	0.00	0.00	0.00	0.00
112-39-0	Methyl palmitate	D14	1786.63	148.20±1.17^{c}	184.98±3.42^{a}	173.98±1.17^{b}	2000	0.07	0.09	0.09	0.00
58130-58-8	6,10-Dodecadien-1-ol,3,7,11-trimethyl-, 1-acetate, (6E)-	D15	2034.03	155.53±1.00^{b}	165.70±0.41^{a}	154.49±0.66^{b}	—	—	—	—	0.00
95-63-6	1,2,4-Trimethylbenzene	E1	1495.58	29.10±1.02^{a}	18.38±0.02^{b}	7.92±0.06^{c}	260	0.11	0.07	0.03	0.00
21693-51-6	1,5,8-Trimethyltetraline	E2	1828.88	20.28±0.87^{a}	17.94±0.56^{b}	5.87±0.02^{c}	—	—	—	—	0.02
475-03-6	1,1,6-Trimethyltetralin	E3	1851.74	32.55±0.30^{a}	29.58±0.22^{b}	8.15±0.09^{c}	20	1.63	1.48	0.41	0.00
30364-38-6	1,1,6-trimethylnaphthalene-1,2-dihydride	E4	1867.54	46.29±1.71^{a}	48.00±0.50^{a}	18.65±0.31^{b}	2.5	18.52	19.20	7.46	0.17
581-42-0	2,6-Dimethylnaphthalene	E5	1724.06	791.67±0.05a^{b}	791.53±0.21^{b}	791.97±0.08^{a}	10	79.17	79.15	79.20	0.32
575-41-7	1,3-Dimethylnaphthalene	E6	2046.39	798.39±0.68^{a}	797.14±0.51^{a}	794.43±0.24^{b}	42	19.01	18.98	18.92	0.06
91-57-6	2-Methylnaphthalene	E7	1687.49	23.68±1.55	22.60±0.19	22.12±0.37	3	7.89	7.53	7.37	0.30
483-78-3	Cadalene	E8	1791.94	161.07±0.03^{b}	161.94±0.14^{a}	148.69±0.05^{c}	—	—	—	—	0.00
35796	Furfural	F1	1140.42	2110.33±40.15^{a}	1368.00±60.97^{b}	715.66±20.53^{c}	—	—	—	—	0.00
124-19-6	1-Nonanal	F2	1111.01	19.61±11.73	23.70±0.10	25.09±0.44	5.8	3.38	4.09	4.33	0.58
78-70-6	Linalool	G1	2226.96	60.77±1.50^{a}	47.42±0.66^{c}	51.72±0.62^{b}	6	10.13	7.90	8.62	0.00
23726-93-4	beta-damascenone	G2	1680.05	21.56±0.21^{b}	25.64±0.20^{a}	15.75±0.55^{c}	0.002	10780.00	12820.00	7875.00	0.00
7212-44-4	Nerolidol	G3	1748.55	798.39±0.68^{a}	153.63±0.33^{b}	148.61±0.04^{c}	2250	0.35	0.07	0.07	0.00
30316-36-0	1,2,3,4-tetrahydro-1,6,8-trimethylbenzene	G4	1827.86	22.07±0.30^{a}	19.39±0.29^{a}	7.61±0.06^{b}	—	—	—	—	0.02
483-76-1	(+)-delta-cadinene	G5	1951.03	145.54±0.03	145.42±0.05	145.24±0.08	—	—	—	—	0.54
39029-41-9	Gamma-terpinene	G6	1619.39	145.13±0.08	145.16±0.04	145.23±0.08	—	—	—	—	0.71
24406-05-1	α-cadinene	G7	1979.1	145.34±0.01^{a}	145.31±0.17^{b}	145.49±0.26^{c}	—	—	—	—	0.00
106-28-5	E,E)-Farnesol	G8	2054.61	146.81±0.10^{C}	147.40±0.07^{b}	149.27±0.22^{a}	—	—	—	—	0.00

10208-80-7	α-muurolene	G9	1941.27	145.33±0.04[b]	145.45±0.16a[b]	145.61±0.09[a]	—	—	—	—	0.28
21391-99-1	α-calacorene	G10	1704.04	181.03±0.06[C]	177.46±0.68[b]	150.84±0.39[a]	—	—	—	—	0.00
20129-39-9	α-corocalene	G11	1885.14	153.63±0.82[C]	152.05±0.44[b]	145.93±0.06[a]	—	—	—	—	0.04
124-07-2	Octanoic acid	H1	2312.48	216.19±1.77[b]	21171.70±3252.86[a]	25074.06±4619.07[a]	15000	0.01	1.41	1.67	0.00
4128-17-0	Farnesyl acetate	H2	1796.99	157.58±1.26[c]	193.26±0.99[a]	176.76±3.21[b]	—	—	—	—	0.00
334-48-5	Decanoic acid	H3	2348.6	3955.92±907.48[c]	14326.24±2274.14[b]	21070.41±3584.32[a]	8000	0.49	1.79	2.63	0.00
14436-32-9	9-decenoic acid	H4	1476	1324.09±9.40[b]	1590.03±44.59[a]	1622.94±62.89[a]	4300	0.31	0.37	0.38	0.00
96-76-4	2,4-Di-tert-butylphenol	I1	1475.65	2376.53±47.53[b]	6384.31±75.05[a]	6821.96±685.58[a]	—	—	—	—	0.00

Note: The threshold represents the threshold acquired in the references [13]; "–" represents undetected.

https://doi.org/10.3390/foods15173004

Table S5. Key differential volatile aroma compounds screened by OAV > 1, VIP > 1, and $p < 0.05$.

CAS	Name	RI	Threshold (μg/L)	VIP
78-83-1	2-Methyl-1-propanol	1480.17	1300	1.07
123-51-3	3-Methyl-1-butanol	2063.55	6500	1.13
111-70-6	1-Heptanol	2157.85	2450	1.13
142-92-7	Hexyl acetate	2080.45	670	1.03
112-17-4	N-decyl-acetate	1566.79	225	1.04
103-45-7	Phenethyl acetate	2250.29	650	1.09
105-54-4	Ethyl butyrate	1032.04	150	1.10
123-66-0	Ethyl Hexanoate	2070.88	76	1.10
106-32-1	Ethyl caprylate	916.77	240	1.06
78-70-6	Linalool	2226.96	6	1.10
23726-93-4	beta-damascenone	1680.05	0.002	1.05
124-07-2	Octanoic acid	2312.48	15000	1.01

https://doi.org/10.3390/foods15173004
